# Cost and clinical outcomes of epilepsy surgery in a Brazilian public hospital: a health economic evaluation

**DOI:** 10.1055/s-0046-1827047

**Published:** 2026-08-13

**Authors:** Raíssa Mansilla Cabrera Rodrigues, Tayla Taynan Romão Bastos, Lizen Clare André Moreira, Vânia de Matos Fonseca, José Alberto Landeiro, Bruno Lima Pessôa

**Affiliations:** 1 Universidade Federal Fluminense, Faculdade de Medicina, Departamento de Neurocirurgia, Niterói RJ, Brazil.; 2 Universidade Federal Fluminense, Faculdade de Medicina, Departamento de Neurologia, Niterói RJ, Brazil.; 3 Universidade Federal Fluminense, Faculdade de Medicina, Niterói RJ, Brazil.; 4 Fundação Oswaldo Cruz, Instituto Nacional de Saúde da Mulher, da Criança e do Adolescente Fernandes Figueira, Rio de Janeiro RJ, Brazil.

**Keywords:** Drug-Resistant Epilepsy, Cost-Benefit Analysis, Health Care Economics and Organizations, Cost-Effectiveness Analysis, Public Health Practice

## Abstract

**Background:**

Epilepsy surgery is an established therapy for drug-resistant epilepsy, but it remains underused in Brazil. Assessing direct costs and outcomes is key for planning in low- and middle-income countries (LMICs).

**Objective:**

To estimate surgery costs in a Brazilian public hospital and evaluate seizure and medication outcomes.

**Methods:**

A retrospective economic evaluation was conducted among patients aged ≥ 16 years who underwent epilepsy surgery at a Brazilian tertiary public hospital between 2014 and 2019. A micro-costing (bottom-up) approach from the healthcare provider perspective was used to estimate actual resource consumption based on hospital administrative records. Costs were stratified by surgical technique, and clinical outcomes were assessed using seizure frequency, antiseizure medication use, and standardized Engel and International League Against Epilepsy (ILAE) classifications.

**Results:**

Twenty patients were included, predominantly with temporal lobe epilepsy, and were mainly treated with resective procedures. Average direct costs varied according to the surgical technique, ranging from approximately US$ 4,200 for resective procedures to US$ 6,800 for disconnective surgeries. Hospital length of stay was the primary cost driver, followed by expenditures on materials and human resources. Overall, the observed costs were proportional to the clinical benefits, as most patients demonstrated favorable seizure control at 2-year follow-up, with consistent reductions in seizure frequency and antiseizure medication use.

**Conclusion:**

The current study presents a transparent hospital-based micro-costing approach to estimate the direct costs of epilepsy surgery in a tertiary public setting of a middle-income country. By focusing on actual resource consumption and standardized clinical outcomes, it supports the clinical effectiveness and economic plausibility of epilepsy surgery within the Brazilian Public Healthcare System and informs future economic evaluations in similar contexts.

## INTRODUCTION

Approximately 70 million people worldwide live with epilepsy, with around 80% of them residing in low- and middle-income countries (LMICs). 1 In Brazil, the prevalence rates range from 9.2 to 18.6 per 1,000 individuals. 2 3 4 5 6 While most patients manage their condition with antiseizure medications (ASMs), approximately 30 to 40% are refractory, making them potential candidates for surgical treatment. 7 8 9 10

Despite high-level evidence demonstrating the effectiveness and safety of epilepsy surgery for a subset of patients with drug-resistant epilepsy, the procedure remains underutilized, particularly in LMICs. 9 11 12 13 14 15 According to the World Health Organization, epilepsy surgery is available in only 41% of countries globally, and in no more than half of the countries in the Americas. 16

This restricted access contributes to prolonged delays in treatment, negatively affecting quality of life, leading to poorer seizure control, increasing the risk of sudden unexpected death in epilepsy (SUDEP), and imposing a substantial economic burden. Indeed, a recent study showed that, on average, 16.7 years elapse between the diagnosis of epilepsy and the initiation of surgical therapy. 17 This treatment gap is associated with a 4- to 9-fold increase in hospital care utilization and a 2.1- to 10.6-fold rise in the overall costs of the disease. 16

In Brazil, however, evidence regarding the direct costs and clinical benefits of epilepsy surgery remains limited. Although resective epilepsy surgery involves substantial upfront costs, studies indicate that it becomes cost-effective within 9 to 10 years after the intervention, or even earlier when indirect cost savings are taken into account. 18 In addition, referral for surgical evaluation is likely to be cost-effective even when the probability of a patient being eligible for surgery is relatively low (∼ 5%). 19

In this context, accurate estimates of economic costs are essential for planning effective treatment strategies, optimizing resource allocation, and assessing the impact of preventive measures. 20 Therefore, the aim of the present study was to estimate the direct costs of epilepsy surgery in a Brazilian tertiary public hospital, evaluate surgical outcomes in terms of seizure control and antiseizure medication use, and compare these findings with international reports.

## METHODS

### Health economic analysis

The current study involved a retrospective cost analysis conducted at a public epilepsy center in the city of Niterói, state of Rio de Janeiro, Brazil. Secondary data sources included patient medical records, the Brazilian Procedure Table Management System (SIGTAP, from the Portuguese Sistema de Gerenciamento da Tabela de Procedimentos) , and hospital bidding processes. All costs were converted to USD (using an exchange rate of 4.04 BRL per 1 USD), and the analysis adhered to the Brazilian Ministry of Health's Methodological Guidelines for Economic Assessment and the Consolidated Health Economic Evaluation Reporting Standards (CHEERS) for reporting. 21

### Study population

The study included patients older than 16 who underwent 1 of 3 epilepsy surgeries: lesionectomy, anterior temporal lobectomy with amygdalohippocampectomy (ATL + AH), or anterior corpus callosotomy. Patients with incomplete medical records were excluded. The analysis was performed from the healthcare payer's perspective, reflecting the Brazilian Public Healthcare context.

### Time horizon and discount rate

The time horizon for cost and outcome measurement was 5 years (2014–2019), chosen to capture relevant long-term consequences. Future costs were adjusted using a 5% annual discount rate, as recommended by the Brazilian Ministry of Health.

### Outcome measures and data collection

Direct costs included expenditures related to human resources, equipment, anesthetics, consumables, and specialized materials, such as orthotics, prosthetics, and neuronavigation systems. All cost information was obtained directly from the tertiary public center where the study was conducted, using internal institutional administrative records and hospital financial reports. These data reflect the actual cost of service provision (resource consumption), rather than reimbursement rates or payments made by third parties.

Specifically, personnel costs were estimated based on the effective working hours dedicated to each surgical procedure, using institutional salary scales in force during the study period, including corresponding payroll charges. Equipment costs were calculated using a linear depreciation method, taking into account the acquisition value recorded in the hospital's administrative procurement processes, the estimated useful life of each device, and the proportional time of use per procedure. Costs related to anesthetics, consumables, and specialized materials were measured based on the actual consumption recorded in surgical charts and institutional inventory control systems, with unit prices derived from hospital bidding and procurement processes.

Therefore, the costs reported in the current study represent a detailed estimate of healthcare resource consumption from the healthcare provider perspective within the Brazilian Public Healthcare System, rather than billing amounts or financial transfers. Detailed calculations related to equipment depreciation, hourly staff costs, and consumable cost categories are provided in Tables 1 2 3 , ensuring methodological transparency and reproducibility.

**Table 1:** Demographic and surgical details of the sample

Sample data	Value
Female sex: n (%)	11 (55)
Mean age at surgery (years)	36 ± 14.34
Mean age at onset of seizures (years)	14.7 ± 8.87
Mean duration of epilepsy (years)	21.1 ± 16.19
Type of epilepsy/ILAE classification 2017: n (%)	
* Focal epilepsy*	18 (90)
* Generalized epilepsy*	1 (5)
* Focal and generalized epilepsy combined*	1 (5)
Temporal lobe epilepsy side: n (%)	
* Right*	11 (73)
* Left*	4 (27)
Surgery performed	n (%)
* ATL* *+* *AH*	13 (65)
* CC*	2 (10)
* LWo/IOM*	5 (25)
Neuronavigation	n (%)
* Used in surgery*	12 (60%)
Complications	n (%)
* Quadrantanopia*	1 (5)
* Psychiatric disorders*	2 (10)
* Surgical site infections*	2 (10)
* Deep vein thrombosis*	1 (5)
Length of hospital stay (days)	
* Mean ward stay*	5 ± 2.28
* Mean ICU stay*	3 ± 1.12
* Mean length of hospital stay*	8 ± 2.89
Histopathological findings	n (%)
Hippocampal sclerosis*	2 (10)*
Hippocampal sclerosis + focal cortical dysplasia*	5 (25)*
* Focal cortical dysplasia*	2 (10)
* Focal cortical dysplasia + Focal lesion or gliosis*	5 (25)
Gliosis without hippocampal sclerosis*	3 (15)*
* No lesion in the submitted sample*	3 (15)

Abbreviations: ATL + AH, anterior temporal lobectomy with amygdalohippocampectomy; CC, corpus callosotomy; ICU, Intensive Care Unit; ILAE, International League Against Epilepsy; LWo/IOM, lesionectomy without intraoperative monitoring; n, number of participants included; SD, standard deviation.

Note: *All cases were clinically and radiologically diagnosed with hippocampal sclerosis.

**Table 2:** Total costs per surgical procedure

Procedure	CC	ATL + AH	LWo/IOM	*p* -value
*Professionals*				
Medical staff	$931.07	$780.37	$739.55	< 0.01
Non-medical staff	$208.71	$208.71	$208.71	
*Hospital Materials*				
Anesthetic material	$349.88	$281.13	$369.06	0.069
Consumable materials	$208.96	$244.69	$175.74	0.076
Equipment	$214.99	$210.95	$210.17	0.921
Sterilization	$8.39	$8.39	$8.39	−
Electricity	$13.04	$9.83	$9.68	−
DMEPOS	$388.61	$388.61	$388.61	−
Neuronavigation	$1,715.18	$1,715.18	$1,715.18	−
*Daily Rates*				
Ward	$932.18	$745.74	$932.18	0.521
ICU	$1,829.51	$1,372.13	$1,372.13	0.327
Total Cost without neuronavigation	**$5,086.34**	**$4,251.29**	**$4,415.75**	**0.318**
Total Cost with neuronavigation	**$6,802.51**	**$5,967.47**	**$6,131.92**	**0.318**

Abbreviations: ATL + AH, anterior temporal lobectomy with amygdalohippocampectomy; CC, corpus callosotomy; DMEPOS, Durable Medical Equipment, Prosthetics, Orthotics, and Supplies; ICU, Intensive Care Unit; LWo/IOM, lesionectomy without intraoperative monitoring.

Notes: All values were converted from Brazilian Real (BRL) to United States Dollar (USD) using the 2019 exchange rate. Due to rounding to two decimal places, the summed totals may not match exactly, but the individual conversions remain accurate to the original BRL values.

**Table 3:** Mean seizure frequency and mean ASM use in the pre- and postoperative periods

Sample Data	CC	ATL + AH	LWo/IOM	All
*Crisis per month*				
Preop	154 ± 206.48	36.9 ± 64.61	37 ± 48.3	49 ± 81.68
Postop (2 years)	5 ± 4.24	8.0 ± 27.64	3.2 ± 5.22	7 ± 22.23
* p* -value	*0.180*	*0.001*	*0.042*	*0.001*
*ASMs*				
Preop	3.5 ± 0.71	2.2 ± 0.69	2.6 ± 1.14	2.4 ± 0.88
* p* -value	0.001	0.607	0.003	0.039
Postop (1 year)	3.0 ± 0.00	1.6 ± 0.65	2.4 ± 1.34	1.9 ± 0.94
* p* -value	−	0.150	0.173	*0.119*
Postop (2 years)	3.5 ± 0.71	1.5 ± 0.78	1.6 ± 0.55	1.7 ± 0.92
* p* -value	−	*0.095*	*1.000*	*0.022*
*SFS*				
Preop	9.5 ± 2.12	8.4 ± 1.16	7.6 ± 0.89	8.3 ± 1.22
Postop (2 years)	5.0 ± 1.14	2.1 ± 1.59	1.6 ± 1.14	2.35 ± 1.76
* p* -value	0.180	0.001	0.039	0.000

Abbreviations: ASMs, anti-seizure medications; ATL + AH, anterior temporal lobectomy with amygdalohippocampectomy; CC, corpus callosotomy; LWo/IOM, lesionectomy without intraoperative monitoring; Postop, postoperative period; Preop, preoperative period; SFS, seizure frequency scale.

Clinical outcomes included monthly seizure frequency, changes in antiseizure medication (ASM) dosages, and surgical effectiveness based on the International League Against Epilepsy (ILAE) outcome classification (classes 1–4).

### Data analysis and sensitivity testing

All statistical analyses were conducted using IBM SPSS Statistics for Windows (IBM Corp.), version 20.0 and Microsoft Excel 2016 (Microsoft Corp.). Normality was tested with the Shapiro-Wilk and Kolmogorov-Smirnov tests. For nonparametric comparisons, the Mann-Whitney and Kruskal-Wallis tests were applied to independent groups, while the Wilcoxon and Friedman tests were used for paired groups. Values of *p* were estimated using bootstrap resampling (1,000 samples), with significance set at *p* < 0.05. Multiple comparisons were adjusted with the Bonferroni method. Categorical data were analyzed with Chi-squared or Fisher's exact test, also supported by bootstrap resampling.

Sensitivity testing was performed to evaluate the robustness of the model under uncertainty. Univariate analysis was conducted using a Tornado diagram, in which the most influential parameters were identified through predetermined minimum and maximum values. Probabilistic sensitivity analysis was conducted via Monte Carlo simulations with 100 iterations, assuming variable independence. In this framework, a Poisson distribution was applied to account for rare and unexpected variations. Parameters such as surgery duration, anesthetic consumption, and the use of surgical materials were modeled through these simulations.

The results of the sensitivity analyses were interpreted by assessing the dispersion of simulated scenarios and calculating standard deviations as measures of uncertainty. Together, these methods provided a comprehensive exploration of parameter variability and confirmed the stability of the model. The analyses adhered to recommendations from the Brazilian Ministry of Health's guidelines and reflected international standards for health economic evaluations.

## RESULTS

### Demographic and surgical data

A total of 20 patients were included, with a female-to-male ratio of 1.2:1, a mean age of 36 years (± 14.34), and a mean epilepsy duration of 21.1 years (± 16.19). Focal epilepsy accounted for 90% of cases, predominantly temporal lobe epilepsy (75%). The most frequent surgical intervention was ATL + AH (65%), followed by lesionectomy (25%) and callosotomy (10%), with neuronavigation applied in 60% of procedures. Among patients with clinical-radiological evidence of hippocampal sclerosis (HS, 55%), histopathology confirmed HS in 7 (35%) and gliosis without HS in 3 (15%). Overall, focal cortical dysplasia was the most frequent histopathological finding (60%). Postoperative complications occurred in 30% of patients, but no surgery-related deaths were observed. Demographics of the sample are summarized in Table 1 .

### Direct cost analysis and comparison

As one of the objectives of the present study was to estimate the average direct costs of epilepsy surgery in a Brazilian tertiary public hospital, we found that these costs amounted to US$ 4,584.46 for procedures performed without neuronavigation and US$ 6,300.63 when this technology was employed. Among procedures, callosotomy was the most expensive (US$ 5,086.34–6,802.51), followed by lesionectomy (US$ 4,415.75–6,131.92) and ATL + AH (US$ 4,251.29–5,967.47). Surgical cost details of the sample are shown in Table 2 .

**Figure 1:**
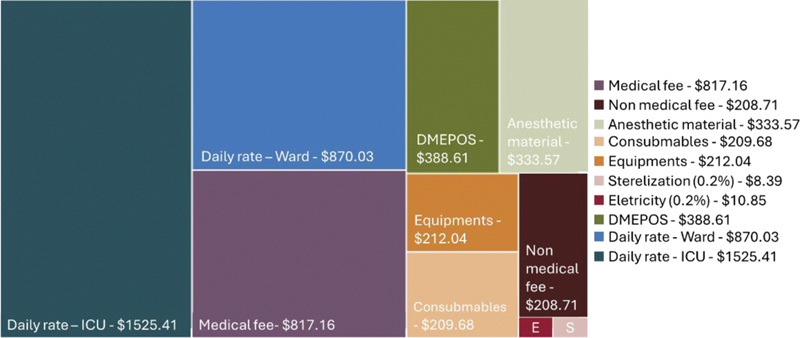
Abbreviations: DMEPOS, Durable Medical Equipment, Prosthetics, Orthotics, and Supplies; ICU, intensive care unit. Note: All values were obtained in Brazilian Real (BRL) of 2019 and converted to United States Dollar (USD) using the 2019 exchange rate. Composition of average costs associated with epilepsy surgery by category.

Daily hospital fees were the main expense (44–49% of the total), followed by hospital supplies and professional fees. Supplies were dominated by durable medical equipment and prosthetics (DMEPOS), which averaged US$ 388.61 per procedure, while anesthetic materials represented the second-largest supply cost, ranging from US$ 281.13 to US$ 369.06. Neuronavigation added a fixed US$ 1,715.18 per case. Professional costs, including medical and non-medical staff, ranged from US$ 739.55 to US$ 931.07, with no statistically significant differences between procedures ( *p* < 0.001). A detailed breakdown of these cost components is illustrated in Figure 1 .

**Figure 2:**
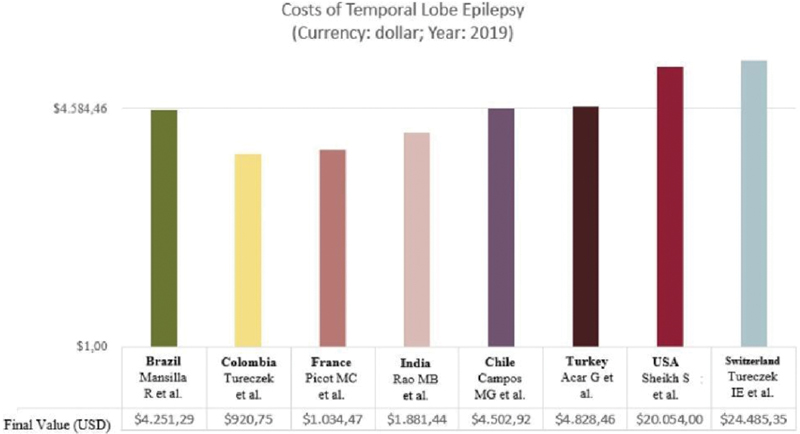
Costs of temporal lobe epilepsy around the world.

The costs of TLE surgery and their proportion relative to GDP per capita across different countries are shown in Figures 2 3 . The direct costs varied widely, ranging from US$ 920.75 in Colombia to US$ 24,485 in Switzerland. When adjusted as a percentage of GDP per capita, Brazil presented an intermediate burden (48%), higher than the United States (31%) and Switzerland (29%), but lower than India (90%) and Türkiye (53%). These findings highlight significant disparities in affordability across countries.

**Figure 3:**
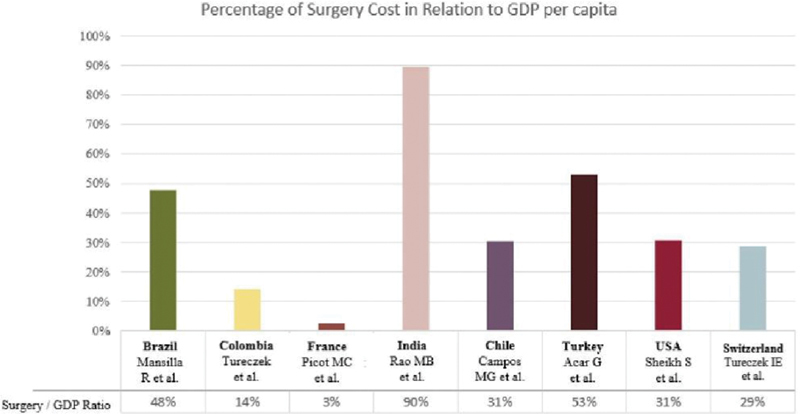
Abbreviation: GDP, gross domestic product. Percentage of the surgery cost relative to GDP per capita among different countries.

### Postoperative outcomes

**Figure 4:**
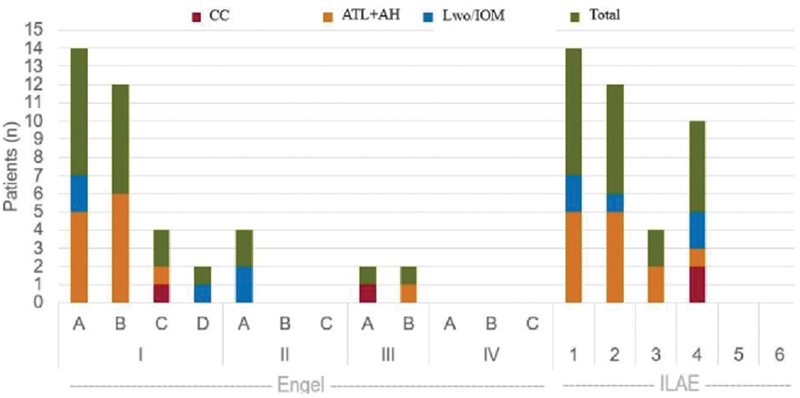
Surgical Outcomes in Relation to Seizures

According to the Engel Outcome Classification, 35% of patients were classified as IA (completely seizure-free), 30% as IB (completely seizure-free), 15% as IC (completely seizure-free), 15% as IIC (completely seizure-free), 20% as IIA (completely seizure-free), and 5% as IIIB (completely seizure-free), with no patients in the other categories. According to the ILAE outcome scale, 35% were classified as class 1, 30% as class 2, 10% as class 3, and 25% as class 4, as shown in Figure 4 .

In addition, as proposed in the present study, surgical outcomes in terms of seizure control were assessed 2 years after surgery. It was observed that 65% of patients achieved an ILAE 1 to 2 classification and 80% reached Engel class I. The mean monthly seizure frequency decreased significantly, from 49 in the preoperative period to 7 postoperatively ( *p* = 0.001), with statistically significant improvements observed in patients undergoing ATL + AH ( *p* < 0.001) and lesionectomy ( *p* = 0.042). These findings were confirmed by the non-parametric Wilcoxon test ( *p* = 0.001). Callosotomy, however, did not reach statistical significance, likely due to the small sample size.

### Antiseizure medication usage and cost reductions

A significant reduction in the use of ASMs was observed in the second postoperative year (p = 0.022), whereas changes in the first year did not reach statistical significance ( *p* = 0.119), a finding that has been previously insufficiently clarified in the literature. Considering all types of procedures, ASM use showed at least one significant modification when comparing the pre- and postoperative periods ( *p* = 0.001). Seizure Frequency Scale (SFS) scores also dropped significantly for ATL + AH ( *p* = 0.001) and lesionectomy ( *p* = 0.039), but not for callosotomy ( *p* = 0.180). Two years after surgery, monthly seizures were reduced by 87.45% in callosotomy cases (CI: 79.01–92.22; 95%), 78.25% for ATL + AH (CI: 68.92–84.99), and 91.35% for lesionectomy (CI: 84.26–95.47), as shown in Table 3 .

**Figure 5:**
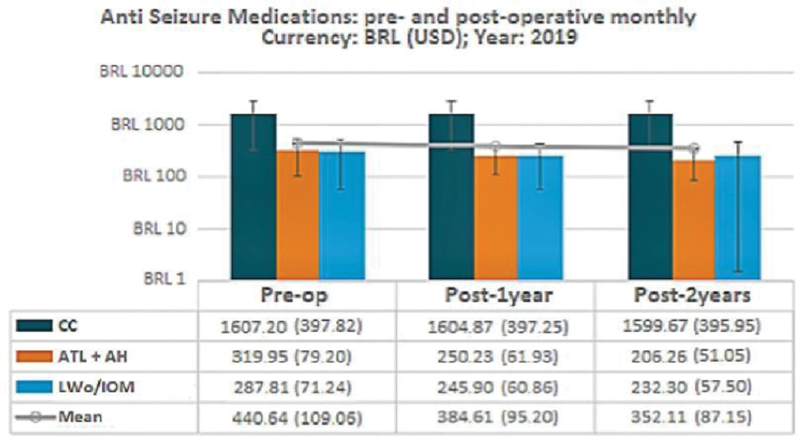
Abbreviations: CC, corpus callosotomy; ATL + AH, anterior temporal lobectomy with amygdalohippocampectomy; LWo/IOM, lesionectomy without intraoperative monitoring; Pre-op, preoperative period; Post-1 year, one year after surgery; Post-2 years, two years after surgery. Note: All values were converted from Brazilian Real (BRL) to United States Dollar (USD) using the 2019 exchange rate. Direct costs of anticonvulsant drugs before and over two years after surgery.

Preoperative ASM costs averaged US$ 109.06 ± $132.37 per month, with a range from US$ 14.84 to US$ 596.11. One-year postsurgery, this average decreased to US$ 95.18, further reducing to US$ 87.10 ± 32.72 in the second year, with costs ranging from zero to US$ 617.47. For ATL + AH, preoperative ASM expenses averaged US 79.20 ± 53.81, dropping to US$ 61.92 ± 34.25 in the first year and US$ 51.05 ± 29.89 in the second year. Lesionectomy patients experienced a reduction in ASM costs from US$ 71.23 ± 57.21 presurgery to US$ 60.89 ± $ 46.50 in the first year and US$ 57.49 ± 57.14 in the second year, as shown in Figure 5 .

In summary, epilepsy surgery showed an average direct cost of US$ 4,584.46 without neuronavigation and US$ 6,300.63 with its use, with hospital stay representing the main cost component. When adjusted for GDP per capita, Brazil demonstrated an intermediate economic burden in international comparison. After 2 years, satisfactory seizure control was observed, with 65% of patients classified as ILAE 1 to 2 and 80% as Engel class I, along with significant reductions in seizure frequency and antiseizure medication use. These findings support that epilepsy surgery in a Brazilian tertiary public hospital is clinically effective and economically reasonable compared with international data.

## DISCUSSION

The present study represents the first direct cost analysis of resective epilepsy surgery in Brazil using a bottom-up approach. The cost of TLE surgery in our study (US$ 4,521.29) is comparable to findings in Chile 22 23 and Türkiye, 24 yet it is approximately twice the cost of the same surgery in India 25 and four times higher than in France 17 and Colombia. 26 In contrast, surgical expenses in Brazil are significantly lower than those in the United States 18 and Switzerland, 26 where costs are 4.7 and 5.8 times higher, respectively. When comparing TLE surgery costs to GDP per capita globally, 22 we observe that the cost in Brazil represents 48% of the average income. This highlights the substantial economic burden of epilepsy surgery in Brazil, particularly given the decline in GDP per capita post-pandemic, from US$ 8,897.49 in 2019 to US$ 6,796.84 in 2020. 22 27

These disparities are explained by differences in salary scales, health technology adoption, and hospital management models. In our sample, the mean direct for all surgeries ranged from US$ 4,585.46 to US$ 6,300.63 per procedure, with hospital daily fees emerging as the primary cost driver. This indicates that organizational efficiency may influence total expenses more than the surgical technique itself or the use of neuronavigation. Importantly, our data suggest that reducing the length of stay by 3 days could save nearly US$ 920 per surgery—approximately 20% of the total cost. Such findings are particularly relevant for both public and private health systems, in which systemic delays often prolong hospitalization and generate avoidable expenditures.

Despite the high upfront costs, epilepsy surgery proved to be cost-effective in our cohort, with 65% and 80% of patients achieving favorable outcomes based on ILAE and Engel classifications, respectively. Beyond direct savings from reduced ASM dependency—annual costs decreased by nearly 20% postsurgery—the marked reduction in seizure frequency (from 49–7 monthly episodes) translated into improved quality of life, facilitating social and workforce reintegration, and generating indirect economic benefits not captured in the current analysis. These findings align with global studies demonstrating that epilepsy surgery remains economically advantageous over the long term. 29 30 31

Medication expenditures further illustrate the surgery's economic impact. Antiseizure medication costs represent 50 to 78.7% of epilepsy-related medical expenses. Before surgery, annual ASM costs for our cohort averaged US$ 1,305.94—equivalent to 14.6% of Brazil's 2019 GDP per capita, 22 markedly higher than global averages. This burden is amplified by Brazil's 32% medication tax rate, compared to a global average of 6%. Following surgery, ASM costs decreased to US$ 1,045.87 annually, reflecting reduced dependency and lowering long-term expenditures.

Overall, the 2-year post-surgery cost of US$ 6,772.74 (27,361.90 BRL) corresponds to an investment of US$ 161.25 (651.47 BRL) per monthly seizure avoided. This metric highlights the high value of surgical intervention, particularly within the LMICs health systems, where resource allocation is critical. Our findings align with those of international studies, thus confirming that, despite initial expenses, epilepsy surgery is a cost-effective intervention with durable clinical and economic benefits. 28 32

In terms of surgical outcomes, patients who became seizure-free or minimally affected typically achieved better quality of life, consistent with ILAE class 1 to 2 and Engel Class I. 33 In our cohort, favorable outcomes were achieved in 72% (ILAE 1–2) and 83% (Engel I), consistent with global reports. 34 35 36 No patient experienced postoperative deterioration, reinforcing the reliability and inter-rater consistency of these classifications. 37 Even among patients who are not fully seizure-free, many reported subjective improvements in quality of life, underscoring that surgical benefits may extend beyond seizure control alone. 33 38 This highlights the value of using seizure frequency as a complementary outcome measure, particularly for palliative procedures such as callosotomy, in which complete seizure control is rarely expected. 38 39 Overall, our findings reinforce that resective epilepsy surgery provides superior outcomes compared to medical therapy alone, 28 consistent with the findings of Picot et al., who reported 74% seizure freedom and a ≥ 50% reduction in seizure frequency in most remaining patients 2 years after surgery. 17

The current analysis highlights key factors influencing surgical outcomes, including surgery duration, available center resources, and surgical expertise. Similar to many studies, our patient sample had an average epilepsy duration of 15 to 20 years before surgical evaluation—a modifiable factor that, if addressed earlier, could lead to improved outcomes. For patients with refractory epilepsy and their families, this study emphasizes the crucial role of the public health system in providing equitable access to surgery. Despite the initial costs, surgery offers significant long-term benefits.

Although the present study has limitations—including its retrospective design, small sample size, single-center setting, and predominance of TLE—it represents the only Brazilian analysis of direct epilepsy surgery costs. By addressing this critical knowledge gap, our findings provide original evidence to inform hospital management strategies and public health policies. Overall, epilepsy surgery in Brazil, despite significant upfront costs, emerges not only as a clinically effective intervention but also as an economically strategic investment for the sustainability of the national health system.

In conclusion, the current study provides a novel economic perspective on epilepsy surgery costs in a middle-income country. By quantifying direct surgical expenses, it establishes a foundation for future cost-effectiveness analyses and long-term evaluations. Such evidence is crucial for aligning the significant upfront costs with the proven clinical benefits of surgery, supporting the sustainability of epilepsy care in LMICs and other resource-constrained health systems.
